# Complete and Draft Genome Sequences of Eight Oceanic *Pseudomonas aeruginosa* Strains

**DOI:** 10.1128/genomeA.01255-17

**Published:** 2017-11-02

**Authors:** Yohei Kumagai, Susumu Yoshizawa, Keiji Nakamura, Yoshitoshi Ogura, Tetsuya Hayashi, Kazuhiro Kogure

**Affiliations:** a Atmosphere and Ocean Research Institute, the University of Tokyo, Chiba, Japan; b Department of Natural Environmental Studies, Graduate School of Frontier Sciences, the University of Tokyo, Chiba, Japan; c Department of Bacteriology, Faculty of Medical Sciences, Kyushu University, Fukuoka, Japan

## Abstract

*Pseudomonas aeruginosa* is one of the most common model bacterial species, and genomes of hundreds of strains of this species have been sequenced to date. However, currently there is only one available genome of an oceanic isolate. Here, we report two complete and six draft genome sequences of *P. aeruginosa* isolates from the open ocean.

## GENOME ANNOUNCEMENT

*Pseudomonas aeruginosa* is a ubiquitous environmental bacterium that is found in diverse natural environments (1) and on macroscopic organisms such as insects, plants, and animals, including humans (2). To date, more than 500 genomes of *P. aeruginosa* strains have been sequenced, which has helped to reveal the evolutional history and adaptation mechanisms of the species (3, 4). However, most strains analyzed so far are host-associated, and few sequences are available for free-living *P. aeruginosa* strains. In particular, only one strain has been sequenced for an ocean-derived *P. aeruginosa* isolate (5). Here, we report eight genome sequences of *P. aeruginosa* strains, isolated from the open ocean, to contribute to a better understanding of the mechanisms of *P. aeruginosa* to adapt to the ocean surface environment.

All eight strains (Ocean-100, Ocean-222, Ocean-238, Ocean-1155, Ocean-1170, Ocean-1175, Ocean-1187, and Ocean-1206) were isolated from the surface layer of the North Pacific Ocean in our previous study (6). Genomic DNA samples were extracted by the standard phenol-chloroform method. To construct genomes of four strains (Ocean-100, Ocean-222, Ocean-1187, and Ocean-1206), the Kapa HyperPlus kit (Kapa Biosystems) was used for library preparation, and paired-end sequences (300 bp of each end) were obtained on a MiSeq instrument with the MiSeq reagent kit version 3 (Illumina). MiSeq reads were assembled using Platanus version 1.2.4 (7) with coverage between 47× and 71×. Genomes of the other four strains (Ocean-1155, Ocean-1170, Ocean-1175, and Ocean-238) were sequenced using the PacBio RS II platform (Pacific Biosciences). PacBio reads were assembled using Hierarchical Genome Assembly Process software (Pacific Biosciences) with coverage between 142× and 230×, followed by manual curation. All sequencing procedures were performed following the manufacturers’ protocols, and all assembly steps were performed using default parameters. Two circular chromosomes, one for Ocean-1155 and the other for Ocean-1175, were obtained. Genomes were annotated using the Prokaryotic Genome Annotation Pipeline at NCBI (8).

The complete and draft genomes had an average length of 6,907,950 bp (ranging from 6,639,630 to 7,067,962 bp) and an average G+C content of 66.03%. No plasmids were detected in the two completely sequenced strains. Notably, the numbers of phage-related genes significantly differed between the strains. For example, Ocean-100 contains 25 phage-related genes and Ocean-222 contains 59. The genome sequences we obtained should provide further insights into the adaptation mechanisms of *P. aeruginosa* to the ocean surface environment.

### Accession number(s).

This whole-genome shotgun project has been deposited at DDBJ/ENA/GenBank under the accession numbers CP022526 (Ocean-1155), CP022525 (Ocean-1175), NMRP00000000 (Ocean-1206), NMRQ00000000 (Ocean-1187), NMRR00000000 (Ocean-222), NMRS00000000 (Ocean-100), NMRT00000000 (Ocean-238), and NMRU00000000 (Ocean-1170).
